# Application of machine learning and deep learning in the diagnosis and treatment of inguinal hernia: a narrative review

**DOI:** 10.3389/fmed.2026.1743178

**Published:** 2026-04-01

**Authors:** Yang Liu, Tian-Hao Xie, Yan Fu, Si-Ning Ha, Xiao-Shi Jin, Xiang-Xiang Ren

**Affiliations:** 1 Department of General Surgery, Affiliated Hospital of Hebei University, Baoding, Hebei, China; 2 Basic Research Key Laboratory of General Surgery for Digital Medicine, Affiliated Hospital of Hebei University, Baoding, Hebei, China; 3 Department of Ophthalmology, Baoding No.1 Central Hospital, Baoding, Hebei, China

**Keywords:** artificial intelligence, deep learning, inguinal hernia, machine learning, review

## Abstract

With the rapid development of artificial intelligence (AI) technology, its application in the diagnosis and treatment of inguinal hernia (IH) has gradually become a research hotspot. As core components of AI, machine learning (ML) and deep learning (DL) demonstrate tremendous potential in medical imaging, disease prediction, and personalized treatment planning. Currently, models developed using ML can effectively predict the risks of postoperative surgical site infection, surgical site occurrence, intestinal resection in incarcerated IH, and postoperative lower extremity venous thromboembolism. DL, as a subset of ML, excels in processing unstructured data such as images and videos. It utilizes deep neural networks to automatically extract data features, thereby enhancing medical image diagnosis and intraoperative navigation capabilities. Studies have shown that DL is highly effective in identifying anatomical landmarks during surgery, which facilitates real-time feedback and surgical training. Generative AI, built on ML theories, shows promise in medical consultations, but its accuracy and reliability require further validation. Overall, ML and DL are revolutionizing the management of IH by improving diagnostic accuracy, optimizing surgical protocols, and enhancing patient outcomes. Future prospects include data integration, real-time feedback, and interdisciplinary collaboration. This article provides a review of the applications of ML and DL in the diagnosis and treatment of IH, offering references for clinical practice and technological innovation.

## 1 Introduction

Artificial intelligence (AI) technologies have advanced at an unprecedented pace, emerging as a pivotal force driving transformation in the healthcare sector. As core components of AI, machine learning (ML) and deep learning (DL) technologies leverage their robust capabilities in data processing and pattern recognition, exhibiting tremendous potential in medical imaging diagnosis, disease prediction, and the formulation of personalized treatment plans (1). With the introduction of ML and DL technologies, the diagnosis and treatment of inguinal hernia (IH) are gradually entering a new era of intelligence and precision. This review aims to systematically summarize the current application status of ML and DL in the diagnosis and treatment of IH, exploring the roles and challenges of these technologies in enhancing diagnostic accuracy, optimizing surgical protocols, reducing complication rates, and improving patient outcomes.

This review adopted a narrative review methodology. The literature search was performed across multiple databases, including PubMed, Web of Science, Cochrane Library, Embase, and IEEE Xplore, prior to October 2024. The search terms encompassed “inguinal hernia,” “machine learning,” “deep learning,” “artificial intelligence,” “generative,” “radiomics,” “surgical navigation,” “predictive model,” and their combinations. The inclusion criteria were as follows: original English-language research studies or clinical trials published between 2015 and 2024 that explicitly involved the application of ML/DL in the diagnosis, treatment, prognosis prediction, or surgical navigation of IH. The exclusion criteria included non-hernia-related studies, studies not involving AI technologies, studies solely utilizing traditional statistical methods, conference abstracts, and literature for which the full text was inaccessible. Initially, a total of 312 articles were retrieved. Following screening by titles and abstracts, 48 articles were retained. After full-text reading, a final total of 11 articles were included in the analysis for this review.

## 2 Distinctions and connections between ML and DL

Over the past decade, AI has emerged as a prominent topic of discussion both within and beyond the scientific community, with a wealth of articles in technical and non-technical journals addressing AI, ML, and DL. However, confusion persists surrounding the terms AI, ML, and DL. While highly interrelated, these three concepts are not interchangeable. AI is defined as “the scientific and engineering field dedicated to creating intelligent machines,” encompassing a diverse range of technologies aimed at enabling machines to emulate human cognitive functions, such as learning, problem-solving, and decision-making (2). ML and DL represent specific methodologies for achieving this objective.

ML can learn from existing data in a straightforward manner, generalize its findings, and infer future, yet unknown, data, thereby enabling it to perform tasks without explicit instructions (3). For example, Wu et al. (4) trained ML models using clinical data to predict the risks of surgical site infection (SSI) and surgical site occurrences (SSO) following IH surgery.

Classical ML depends on structured, labeled data for prediction and requires considerable human supervision (5). In contrast, DL, as a subset of ML, does not necessarily require labeled datasets. DL can be regarded as a further specialization of ML. Analogous to the way the human brain processes information and makes decisions through neural pathways, DL utilizes artificial neural networks to simulate this process. These networks are capable of learning incrementally from data, enabling them to make complex inferences as they gather more information. DL can exploit raw data such as text, images, and videos to identify features and distinguish explicit patterns, thereby discovering data groupings without human intervention (6).

The distinction between ML and DL is defined by the number of layers in neural networks, also known as hidden layers (7). When there are more than three hidden layers, the algorithm is considered a DL model. Hidden layers serve as intermediate stages between the input and output of a neural network. They play a crucial role in capturing complex patterns within the data, which makes neural networks highly effective for a variety of applications, such as image and speech recognition, as well as natural language processing. The configuration and weights of these hidden layers are of paramount importance for creating an effective neural network model. In this context, Elhage et al. (8) have demonstrated that a DL architecture utilizing an 8-layer Convolutional Neural Networks (CNNs) can effectively predict the complexity of abdominal wall hernia surgery, as well as surgical outcomes and postoperative complications, through computerized tomography (CT) analysis.

## 3 Applications of ML

ML, as an emerging tool for disease prediction and imaging analysis, has made significant contributions to the healthcare system (9). ML encompasses a variety of models, which can be differentiated based on task types (such as classification, regression, clustering, etc.) and data characteristics (including structured, unstructured, small-sample, large-scale, etc.). Continuous ML methods are capable of providing high accuracy and can ultimately lead to the development of predictive tools that, in some cases, outperform traditional statistical modeling.

IH surgery typically involves clean incisions; nevertheless, the risks of postoperative SSI and SSO still remain. The occurrence of SSI or SSO heightens the risk of postoperative recurrence in patients with IH, imposes a greater financial burden on patients, and places additional strain on social healthcare resources. Therefore, it is of utmost importance to predict the risk of postoperative infection in patients, which allows for the implementation of proactive measures, such as patient education and optimized postoperative care, to prevent infections.

Wu et al. (4) collected data from 491 patients who underwent open IH repair. They utilized five ML methods, namely generalized linear models, random forests, support vector machines, neural networks, and gradient boosting machines, to predict the risks of postoperative SSI and SSO. Their findings indicated that the random forest model demonstrated the best performance, with area under the curve (AUC) values of 0.849 and 0.740 for SSI and SSO predictions, respectively. Furthermore, they developed an online calculator to aid clinicians in risk prediction.^1,2^ They identified diabetes as a significant risk factor and also underscored the limitations of the study and the potential value of the predictive model for personalized treatment.

Incarcerated IH represents an emergency condition that can result in intestinal necrosis, perforation, and intra-abdominal infection, thereby necessitating prompt surgical intervention. Early identification of the risk of intestinal resection is of critical importance for patient management.

Li et al. (10) performed a retrospective analysis of 214 patients with incarcerated IH, randomly allocating them into a training set (161 cases) and a test set (53 cases). From preoperative CT images, they extracted 1,561 radiomic features and, following dimensionality reduction, selected 13 key features (such as the root mean square and maximum value in the first-order statistics of the wavelet_LLL decomposition level, etc.). They constructed a radiomic model using the logistic regression algorithm and combined it with clinical data to form a comprehensive model (radiomic-clinical nomogram). The comprehensive model achieved AUC values of 0.864 and 0.800 in the training and test sets, respectively, significantly outperforming the standalone clinical or radiomic models. It effectively evaluated the risk of intestinal resection in patients with incarcerated IH, providing robust support for clinical decision-making and demonstrating the immense potential of integrating medical imaging with clinical data in enhancing surgical planning and patient management.

Liu et al. (11) developed a ML -based multi-layer and imbalanced classification predictor to aid in diagnosing whether intestinal necrosis has occurred preoperatively in children with IH. The study utilized blood routine and liver and kidney function test data from 3,807 children without intestinal necrosis and 170 children with intestinal necrosis. Missing values were managed using methods such as median, mean, and mode-area random interpolation, and ensemble learning was adopted to address data imbalance. Three models were constructed, based on blood routine, liver and kidney function, and a combination of both, respectively, and predictors were trained using the random forest algorithm. Experiments demonstrated that the performance of the models significantly improved after feature selection. The hybrid model achieved an accuracy of 86.43%, a sensitivity of 84.34%, a specificity of 96.89%, and an AUC value of 0.91. The study underscored the importance of key features such as C-reactive protein in diagnosis, providing clinicians with a valuable auxiliary diagnostic tool while also indicating directions for future improvements.

Yan et al. (12) carried out a study that employed ML techniques to dynamically predict the risk of lower extremity venous thromboembolism (VTE) during the hospitalization period following IH repair. The study incorporated trial data from 11,305 adult patients with hernia across 58 medical institutions. Data imputation was performed using the random forest algorithm, and adaptive synthetic sampling was utilized to balance the data. The study independently screened key preoperative and postoperative predictive variables and applied nine ML models for prediction, including logistic regression, support vector machines, and TabNet, among others. The results indicated that the TabNet model exhibited excellent performance in both the preoperative and combined datasets, achieving receiver operating characteristic (ROC) values of 0.71 and 0.74, respectively. The study identified seven preoperative and ten postoperative key predictors, such as age, history of VTE, family history of thrombosis, type of surgery, and compression time, all of which had a significant impact on the risk of VTE. Additionally, the study put forward clinical decision-making recommendations based on the model results, with the aim of guiding VTE prevention and treatment. In the future, the study will conduct external validation in clinical trials and develop an online risk assessment tool to further improve the predictive accuracy and clinical applicability of VTE risk assessment.

## 4 Application of DL

DL, as a specialized extension of ML, leverages the powerful capability of deep neural networks to automatically extract data features, demonstrating significant advantages in processing large-scale unstructured data (such as images and videos). By employing techniques such as CNNs, Recurrent Neural Networks (RNNs), and Generative Adversarial Networks (GANs), its performance in medical image diagnosis, intraoperative navigation, and other areas can be further enhanced.

The HerniaSurge Group points out that to achieve a high level of proficiency in transabdominal preperitoneal (TAPP) hernia repair, specialized surgeons need to perform 50–100 procedures. However, the recurrence rate of TAPP still fluctuates between 0.2% and 25% (13). Apart from technical and experiential factors, inadequate anatomical understanding of the myopectineal orifice is a significant cause of recurrence (14). Meanwhile, postoperative chronic inguinal pain, as a major complication of IH repair, severely impairs patients’ quality of life. Although the laparoscopic approach has been shown to reduce the risk of chronic pain compared to the anterior approach, its incidence still ranges from 2.6% to 13.8% (14). In most cases, chronic pain stems from nerve injury caused by fixation tacks, followed by improper anatomical placement of the mesh directly on exposed nerve surfaces.

During TAPP procedures, nerves at risk of injury include the lateral femoral cutaneous nerve, the femoral branch of the genitofemoral nerve, and the genitofemoral nerve itself. The “pain triangle” is located below the iliopubic tract and lateral to the testicular vessels, through which branches of the genital and femoral nerves, as well as the lateral femoral cutaneous nerve, pass. The “triangle of doom” is formed by the vas deferens, testicular vessels, and external iliac vessels, and may contain the genital branch of the genitofemoral nerve. Since nerves are often not directly exposed during TAPP, surgeons must rely on anatomical landmarks to identify high-risk areas and avoid complications. Multiple research teams have conducted studies using DL techniques to address these issues and have achieved positive results.

Takeuchi et al. (15) collected 160 TAPP surgical videos and developed an anatomical structure view detector based on a deep neural network. This detector can automatically identify seven key anatomical landmarks: the pubic symphysis, Hesselbach’s triangle, Cooper’s ligament, the iliac vein, the triangle of doom, the internal inguinal ring, and the iliopsoas muscle. After training and validation, the model achieved an average precision of 51.2% in single - image validation. In video validation, it attained an average accuracy of 77.1% and an F - score of 75.4%. The study results show that this AI system can effectively detect the anatomical features of the myopectineal orifice in real - time during surgery, providing immediate feedback to the surgeon performing the operation. This not only helps surgeons execute surgical steps more effectively, enhancing the standardization and safety of the procedure, but also shows promise for surgical training and education. By automatically highlighting anatomical landmarks, it can assist novices in more quickly grasping the concept of the myopectineal orifice and mastering surgical techniques.

Mita et al. (16) developed an AI system named EUREKA, utilizing deep - learning technology. During TAPP surgery, this system has the capability to automatically predict anatomical structures. The average Intersection over Union (IoU) and F1/Dice scores for loose connective tissue, nerves, the vas deferens, and microvessels were 0.33 and 0.50, 0.24 and 0.38, 0.50 and 0.66, and 0.30 and 0.45, respectively. In particular, under appropriate tension, the system is capable of clearly identifying and displaying the layers of loose connective tissue. It also precisely recognizes key nerves, such as the lateral femoral cutaneous nerve and the femoral branch of the genitofemoral nerve, as well as the vas deferens, and effectively marks the distribution of microvessels. The results demonstrate the reliability and training value of this system as a surgical support tool. Moreover, they envisage its potential applications in reducing complications and shortening the learning curve.

Zang et al. (17) carried out an in - depth investigation into the surgical phase recognition technology for AI - based robot - assisted laparoscopic IH repair. In this study, a dataset for model training and evaluation was established by collecting 209 surgical video clips from 8 surgeons, followed by meticulous annotation and preprocessing. Concurrently, a model development competition was organized among master of engineering students, which swiftly validated the performance of various deep - learning models. The baseline study confirmed the efficacy of simple models such as ResNet - 50 in handling such tasks and highlighted the impact of data imbalance on model performance, along with proposing corresponding solutions. Furthermore, the research delved into more advanced model architectures, including Perceiver IO based on the attention mechanism and video swin transformer designed for efficient video classification. These models exhibited outstanding performance in the surgical phase recognition task, attaining an accuracy rate as high as 0.85. This study not only advances the development of automated surgical analysis technology but also underscores the pivotal role of interdisciplinary collaboration in optimizing medical workflows.

Sato and Ishikawa (18) randomly selected 62 cases (comprising 73 hernias) from 188 TAPP surgical cases (involving 222 hernias) for analysis. A total of 3,323 images of the musculopelvic aperture were labeled for training purposes. Utilizing EfficientNetB7 as the core component, a feature pyramid network segmentation model was developed to identify three key anatomical landmarks: the vas deferens, genital vessels, and inferior epigastric vessels. When identifying these structures (the vas deferens, genital vessels, and inferior epigastric vessels), the model attained Dice coefficients of 0.67, 0.68, and 0.70, respectively, with a correct recognition rate of 90%. Through the recognition of these important anatomical structures, the model aids in reducing nerve damage, thereby mitigating the risk of post - operative chronic pain. However, in female patients, when the round ligament of the uterus serves as a substitute for the vas deferens, the model’s detection accuracy declines or it fails to detect the relevant structure.

Ortenzi et al. (19) collected and annotated 619 totally extraperitoneal (TEP) surgical videos. By leveraging DL and computer vision technologies, they trained a high - precision AI model. This model is capable of automatically identifying six key steps in laparoscopic TEP IH repair surgeries, namely balloon dissection, entry into the preperitoneal space, preperitoneal dissection, hernia sac management, mesh placement, and mesh fixation. The overall accuracy rate in the complete surgical process reached 88.8%. Among these steps, the recognition accuracy for hernia sac management was the highest (94.3%), while the accuracy for preperitoneal dissection was relatively lower (72.2%). Meanwhile, the system adapted through a model pre - trained on various general surgical procedures (such as cholecystectomy, appendectomy, and sleeve gastrectomy) to enhance its performance on TEP videos. This study demonstrates the immense potential of AI in surgical video analysis, providing a new tool for the standardization and improvement of surgical quality.

In conclusion, the application of DL in IH repair offers new opportunities and hope for enhancing surgical outcomes, reducing the incidence of complications, and optimizing training and education. With the continuous development and refinement of technology, it is believed that DL will play an even more crucial role in the medical field.

## 5 Application of generative AI

Generative AI models are essentially systems built on machine - learning theories, with the core objective of automatically learning the mapping relationship between inputs and outputs in a data - driven manner (20). Modern generative AI mainly employs the transformer architecture in DL for implementation. This is a type of deep neural network based on the self - attention mechanism. With the widespread adoption of generative AI, the public may use it to inquire about issues related to different medical conditions. However, there is a lack of data indicating whether conversational AI can provide reliable medical information (21).

In a study (22) on the accuracy and appropriateness of ChatGPT’s responses to questions related to IH management, it was found that the inter - rater reliability among five hernia surgery experts was 69.3% (95% confidence interval: 42.6–85.7). Based on clinical experience and international IH guidelines, 22 questions were proposed, covering the definition, diagnosis, and treatment of IH. Only five answers were rated as “appropriate” by all the experts, and just one question was rated as “appropriate” by one expert. The proportion of experts’ ratings on the appropriateness of answers ranged from 54% to 68.2%. At least three experts considered the answers to 68.2% of the questions to be “appropriate.” Seven questions were rated as “inconsistent” by one expert. Regarding specific question - answering: the question about the types of meshes and risks for IH was rated as “inconsistent” by all the experts. ChatGPT failed to mention the fact that most IHs are asymptomatic and did not point out that permanent synthetic meshes are the most commonly used type. When answering the question about the best non - mesh repair method, ChatGPT listed the McVay repair and the use of biological meshes but did not mention robotic surgery. Through the method of expert evaluation, the study conducted an in - depth analysis of ChatGPT’s performance in answering questions about IH management, revealing its limitations and inaccuracies in providing medical information. It emphasizes the need for a cautious attitude when using AI tools for medical consultations.

Another study (23) focused on the application of ChatGPT in addressing controversial issues related to pediatric IH surgery. Through a comparative analysis with the guidelines of the European Association of Pediatric Surgeons, it was found that ChatGPT’s answers were consistent with the guidelines on most issues, but there were also differences, especially in terms of details and the level of evidence. ChatGPT’s responses were in line with the guidelines on issues such as surgical approaches (open or laparoscopic), timing of surgery, and anesthesia methods. However, for aspects like exploring contralateral occult hernias and asymptomatic irreducible hernias, due to high evidence heterogeneity, the guidelines did not provide clear recommendations, while ChatGPT offered evidence - based suggestions. Expert scoring indicated that the overall quality of ChatGPT’s answers was relatively high, but its reliability needed improvement. ChatGPT demonstrated potential in clinical decision - making, yet it also required further validation and optimization. It was recommended to use ChatGPT cautiously in combination with other reliable data sources.

The application of generative AI in the diagnosis and treatment of IH should be strictly limited to serving as an auxiliary tool, such as for patient education or literature summarization. It must not replace clinical judgment or be utilized as an independent basis for decision-making. The primary risks encompass the “hallucination” risk of generating inaccurate information, outdated knowledge, inconsistent responses, and data privacy issues, which require mitigation through rigorous validation, human oversight, and clear policies. Healthcare institutions should establish usage guidelines, clearly identify AI-generated content, and ensure cautious integration into clinical workflows on the premise of compliance and safety.

## 6 Strengths and weaknesses

The studies summarized in Table 1 illustrate the breadth and depth of current ML and DL applications in IHs management. A critical appraisal of their methodological quality and evidence strength reveals several notable strengths. The diversity of clinical scenarios addressed–ranging from preoperative risk prediction of SSI, VTE, and bowel resection, to intraoperative anatomical landmark recognition and surgical phase detection, and even generative AI for patient consultation–underscores the versatility of these technologies across different stages of hernia care. Many investigations employ state-of-the-art algorithms such as random forests, TabNet, EfficientNet, and transformers, and integrate multi-modal data including imaging, radiomics, and clinical variables. Ensemble methods and sophisticated neural architectures are frequently used to boost predictive accuracy. Reported performance metrics are promising, with area under the curve values often exceeding 0.80 and accuracy surpassing 85 percent in several studies, indicating strong discriminative ability within the development cohorts. Moreover, several studies have produced practical outputs such as online risk calculators and real-time surgical feedback systems, which have direct potential to enhance clinical decision-making and surgical training.

**TABLE 1:** Summary of representative applications of AI in the diagnosis and treatment of inguinal hernia.

References	Application scenario	Data type	Model	Sample size	Performance metrics	Validation method	Clinical significance
Wu et al. (4)	Prediction of SSI/SSO risk	Structured clinical data	Random forest and 4 other ML models	491 cases	AUC: 0.849 (SSI), 0.740 (SSO)	Internal validation	Online calculator for personalized risk assessment
Li et al. (10)	Prediction of bowel resection risk in incarcerated hernia	CT radiomics + clinical data	Logistic regression + nomogram	214 cases	AUC: 0.864 (training), 0.800 (test)	Training/test split	Aids preoperative decision-making and surgical planning
Liu et al. (11)	Diagnosis of intestinal necrosis in pediatric hernia	Blood routine + liver/kidney function	Random forest + ensemble learning	3,977 cases	Accuracy 86.43%, AUC 0.91	Cross-validation	Assists preoperative diagnosis and improves pediatric hernia management
Yan et al. (12)	Dynamic prediction of postoperative VTE risk	Pre-/postoperative clinical variables	TabNet, logistic regression, SVM, etc., (9 models)	11,305 cases	ROC: 0.71 (preoperative), 0.74 (combined)	Adaptive synthetic sampling, random forest imputation, internal validation	Identifies key predictors to guide VTE prevention
Takeuchi et al. (15)	Intraoperative identification of anatomical landmarks	Surgical videos	Deep neural network	160 videos	Average accuracy 77.1%, F1 75.4%	Video sequence validation	Real-time navigation enhances surgical safety and education
Mita et al. (16)	Intraoperative tissue and nerve recognition	Surgical videos	Deep learning (EUREKA system)	Not specified	IoU 0.24–0.50, Dice 0.38–0.66	Internal validation	Aids identification of high-risk structures to reduce nerve injury
Sato and Ishikawa (18)	Segmentation of anatomical landmarks	Surgical images	EfficientNetB7 + FPN	62 cases (73 hernias)	Dice 0.67–0.70, recognition rate 90%	Image segmentation evaluation	Assists localization of key structures to reduce chronic postoperative pain
Zang et al. (17)	Surgical phase recognition in robotic-assisted IH repair	Surgical video clips	ResNet-50, Perceiver IO, video swin transformer	209 clips from 8 surgeons	Accuracy up to 0.85	Internal validation (model competition)	Advances automated surgical analysis and workflow optimization
Ortenzi et al. (19)	Surgical phase recognition	TEP surgical videos	Deep learning model	619 videos	Overall accuracy 88.8%	Phase recognition evaluation	Standardization of surgical workflow and quality assessment
Lima et al. (22)	Appropriateness of ChatGPT answers on IH management	Textual Q&A (22 questions)	ChatGPT	22 questions, 5 experts	Inter-rater reliability 69.3%; appropriateness 54%–68.2%	Expert agreement evaluation	Reveals limitations of generative AI in medical consultation; use with caution
Wang et al. (23)	ChatGPT responses to controversial pediatric IH issues	Textual Q&A (comparison with EUPSA guidelines)	ChatGPT	Expert scoring	High consistency with guidelines but differences in detail/evidence level	Expert evaluation	ChatGPT shows potential but needs reliability improvement; combine with trusted sources

^1^ https://wuqian17.shinyapps.io/predictionSSI/

^2^ https://wuqian17.shinyapps.io/predictionSSO/

Nevertheless, important limitations must be acknowledged. The majority of studies are retrospective and based on single-center data, which limits generalizability and may introduce selection bias. Even when multi-center data are used, external validation in independent prospective cohorts is almost universally lacking, raising concerns about overfitting and real-world performance drift. Sample sizes, particularly for DL tasks involving surgical video analysis, are often modest relative to model complexity, potentially affecting stability and robustness. Issues of data imbalance and variability in expert annotations are acknowledged but not always rigorously addressed; few studies report inter-rater reliability or employ techniques to mitigate these biases. Model interpretability receives limited attention, with only a minority of papers discussing feature importance or employing explainability methods, which hinders clinical trust and adoption. The generative AI studies rely on qualitative expert ratings rather than quantitative performance measures, providing low-level evidence that highlights inconsistencies and the need for cautious integration into clinical practice.

Meanwhile, data drift over time can degrade model performance as clinical practices evolve. Continuous monitoring and periodic retraining are necessary but are currently lacking. Class imbalance and label noise affect model reliability, particularly for rare outcomes. Subjective expert annotations introduce further variability that impairs model training and evaluation. Model calibration and interpretability are critical for clinical trust. Poorly calibrated probabilities can mislead clinical decision-making, and black-box systems hinder clinical adoption. These issues remain underexplored.

## 7 Conclusion

Significant progress has been made in the application of ML and DL to the diagnosis and treatment of IHs, providing robust support for early disease detection, risk assessment, prediction and management of postoperative complications, as well as optimization of the surgical process. In the future, with the continuous development of data integration and standardization, multi - modal data fusion, real - time feedback, rigorous external validation and dynamic adjustment, as well as interdisciplinary collaboration and training, the application prospects of ML and DL in the diagnosis and treatment of IHs will be even more promising.
